# Characteristics of Gait Variability in the Elderly While Walking on a Treadmill with Gait Speed Variation

**DOI:** 10.3390/ijerph18094704

**Published:** 2021-04-28

**Authors:** Bohyun Kim, Changhong Youm, Hwayoung Park, Myeounggon Lee, Byungjoo Noh

**Affiliations:** 1 Department of Health Sciences, The Graduate School of Dong-A University, Busan 49315, Korea; 2177638@donga.ac.kr (B.K.); app00113@donga.ac.kr (H.P.); freestyle710@naver.com (M.L.); 2 Department of Health Care and Science, Dong-A University, Busan 49315, Korea; 3 Department of Kinesiology, Jeju National University, Jeju 63243, Korea; bnoh@jejunu.ac.kr

**Keywords:** gait variability, inertial measurement unit, gait stability, spatiotemporal characteristics, elderly individuals, coefficient of variance

## Abstract

Gait variability (GV), which is a variable for predicting mobility issues and risk of falling in elderly people, is defined as the fluctuation in spatiotemporal characteristics from one step to the next in walking. The goal of this study was to analyze the age- and sex-related spatiotemporal variability characteristics of elderly individuals using the measurements taken while walking on a treadmill for one minute based on gait speed variation. Gait testing was conducted on 225 healthy male and female individuals aged 60–79 years who were able to walk and move on their own and, specifically, walk on a treadmill for one minute. The test was performed at three speed conditions—the preferred speed of the participant, 20% higher than the preferred speed, and 20% lower than the preferred speed—and data were recorded using shoe-type data loggers. The different age groups and sex could be distinguished using the coefficient of variance (CV) of the double support phase and gait asymmetry (GA) at the preferred speed, and CVs of stride length and stance phase at faster speed. The results indicated that the values of GV obtained from the test were used to determine the variation in gait characteristics of elderly individuals.

## 1. Introduction

Aging can cause degeneration of the central and peripheral nervous and musculoskeletal systems [1]; particularly, the aging-induced atrophy of the motor cortical regions in the central nervous system can affect balance, coordination, and gait [2]. Impairments, such as decrease in muscle strength and proprioceptive feedback, and degeneration of nerves in the brain and visual, vestibular, and sensory nervous systems can lead to mobility problems that not only increase the risk of falling in the elderly but also affect their gait ability [1,3,4]. It has been reported previously that the prevalence of gait disorders associated with reduced mobility increases rapidly at 70–79 years compared to that in 60–69 years (22.5% increase in males, 30.7% increase in females) [5]. Such gait disorders may also be associated with falls, lower cognitive function, depression, and a diminished quality of life [5].

Gait analysis is used to measure the overall health status of elderly individuals, for instance physical functioning, proneness to falls, and life expectancy and to aid in early diagnosis and monitoring disease progress to improve the efficacy of treatment interventions [6,7]. Recently, the study of gait variability (GV) has increased since they may be more sensitive in quantifying aging and pathologic alterations in the motor control system and obtaining objective measurement variables of mobility and functional status [8]. GV is defined as fluctuations in spatiotemporal characteristics from one step to the next in walking [9], and it reflects the consistency and stability of the gait [10]. A low degree of GV reflects an automatic mechanism that requires minimal attention, and is associated with efficient gait control and safety [11,12,13]. However, high degree of GV leads to increased walking energy cost [14] and decreased balance because of imperfect sensorimotor control due to aging [9]. Therefore, it can be used as a variable to predict mobility problems [15] and risk of falling in elderly individuals [16].

Previous studies on GV with increasing age among the elderly have reported increased CVs of the step width [17,18], step length and time, double support time [18,19], stride length and time, stance time, swing time, and single support time [19]. In addition, previous studies on GV based on the sex of the elderly have reported that CVs of the step width and stride length were significantly higher in females than those in males [20]. However, most previous studies obtain data from participants considering only a few steps on 4–10 m-long walkways, and use the average values in analyses [17,18,19,20]. The accuracy of these results in representing the actual walking patterns in daily life is questionable [21]. Recently, it has been suggested that the reliability of GV measurements can be improved by analyzing at least 30–40 steps of continuous walking [21,22]. Treadmill-based gait analysis has the advantage of reducing the space required relative to over-ground walking assessment [23,24], and enables the acquisition of data for multiple continuous steps while allowing for simplified control of the walking speed [24,25]. Some previous studies have reported that treadmill gait exhibits similar patterns to over-ground gait [23,24,25,26]. Therefore, multiple continuous steps of gait analysis for healthy people with treadmill experience may provide reliable and objective data [27].

Previous studies on GV were conducted using self-preferred speed [17,18,19,20] for which the gait characteristics were optimized via neural and biomechanical mechanisms that minimized sensory feedback control from the highest level of the nervous system. This limited the ability to generalize GV results [28]. Recently, a significant decline in gait ability has been reported, based on the range of the quantitative speed (e.g., ±20% of the individual’s preferred walking speed) [13,29,30]. Hence, gait stability analysis at slower, faster, and self-preferred speeds maybe beneficial in the evaluation of motor control ability and coordination processes simultaneously, and understanding the gait characteristics among the elderly [30,31].

Given the reported advantages of GV research using treadmills, in this study we investigated the age and sex-related spatiotemporal variability characteristics of gait in elderly individuals (over 60 years) walking on a treadmill for one minute at three speed conditions. Therefore, we adopted the following objectives for this study (i) to investigate the differences in GV characteristics among the elderly between 60–69 years and 70–79 years and between males and females, based on data obtained while walking at various speeds, (ii) to identify variables that can distinguish the age and sex-specific differences in GV characteristics among the elderly based on data obtained while walking at various speeds. We hypothesized that GV may significantly differ between 60–69 years and 70–79 years, and also between males and females. In addition, we hypothesized that the variables that can distinguish GV between 60–69 years and 70–79 years and between males and females can be identified using our approach.

## 2. Materials and Methods

### 2.1. Participants

**Figure 1:**
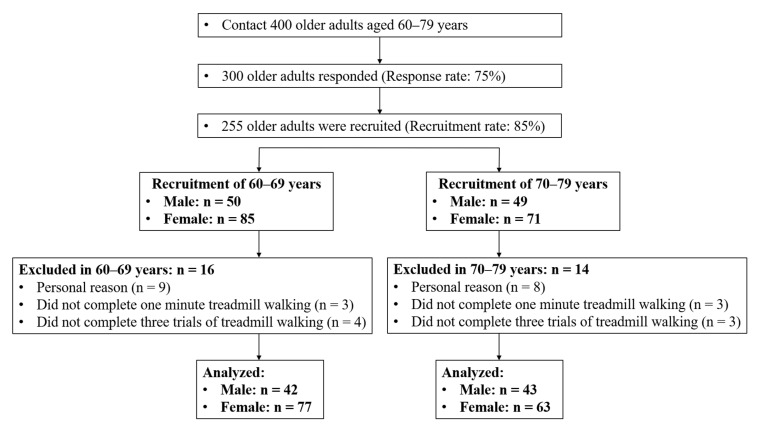
Flow diagram of participant recruitment process.

Participants were finalized based on a community-wide survey conducted in Busan Metropolitan City in 2018. The participants of this study were recruited through announcement, promotion, and direct contact through a public sports facility. We contacted 400 individuals aged between 60 and 79 years that lived in the community and finalized on 300 participants for the study (response rate: 75%). The inclusion criteria for participants were as follows: (1) They could walk and move on their own, (2) they could walk on a treadmill for one minute. Participants with histories of musculoskeletal injuries or neurological problems within six months before this study were excluded, because such issues might affect their gaits. Forty-five participants were excluded according to the inclusion and exclusion criteria. Thirty participants were excluded from the study for the following reasons: 17 did not participate in the test, six did not complete the treadmill walking exercise in one minute, and seven did not complete the treadmill walking trials for three speed conditions. In total, 225 older adults completed three treadmill walking trials at slower, preferred, and faster speed conditions, respectively (Figure 1). All participants read and signed an informed consent document. The study was approved by the Institutional Review Board of Dong-A University (IRB number: 2-104709-AB-N-01-201808-HR-023-02).

### 2.2. Instrumentation

The gait analysis equipment comprised shoe-type data loggers (Smart Balance SB-1^®^, JEIOS, Busan, Korea) with embedded inertial measurement units and a gait analysis system (DynaStab^TM^, JEIOS, Busan, Korea). The shoe-type data loggers featured inertial measurement units (IMU-3000, InvenSence, San Jose, CA, USA) that can measure triaxial accelerations up to ±6 g and triaxial angular velocities up to ±500°/s along three orthogonal axes [32,33]. The inertial measurement units were embedded in the outsole of each shoe and the measured data were transmitted to the gait analysis system using a Bluetooth wireless connection. The shoes were sized to fit each participant. In case of size problems, these were adjusted using additional insoles and Velcro to tie the shoes. A gait analysis treadmill (HK-365, Healthkeeper, INFINITY, Seoul, Korea) was used to adjust the speed from 0.5 to 16 km/h in 0.1 km/h.

### 2.3. Test Procedure

The experimental procedures involved the measurement of demographic characteristics, application of questionnaires, and gait testing, which were performed for one day. Biometric data such as body height, mass, and fat percentage were recorded for each participant, and all the participants completed the questionnaires designed to assess their physical activity levels and cognitive functioning. Physical activity was evaluated using the International Physical Activity Questionnaire-Short Form (IPAQ-SF) that categorizes the physical activity levels as vigorous, moderate, or low. Based on the questionnaire results, we assessed the frequency of activity (days per week) and calculated the corresponding metabolic equivalents (METs) (METs per week) [34]. Cognitive function was assessed using a mini-mental state examination (MMSE) with a total of 30 questions to test factors, such as orientation, attention, memory, language, and visual-spatial skills [35]. Both questionnaires were filled-up voluntarily by the participants.

**Figure 2:**
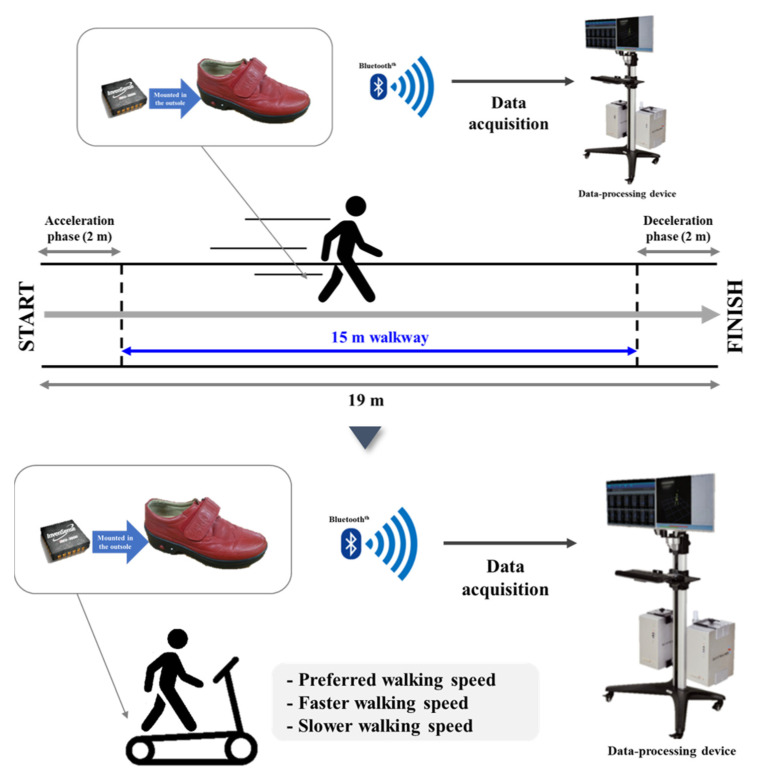
Treadmill walking tests: Preferred speed, 20% faster than preferred speed, and 20% slower than preferred speed.

Before performing the treadmill walking test, each participant performed an overground walking test on a straight 19 m-long walkway to measure their preferred speed, which was calculated by dividing the 15 m length of the section, ±2 m sections for acceleration and deceleration, respectively, by the elapsed time to minimize the error in the average self-preferred speed measurement induced by acceleration and deceleration (distance/walking duration). Each participant then performed treadmill walking adaptation at the preferred walking speed obtained from the overground walking measurement; if the speed difference caused problems with treadmill gait adaptation, the preferred walking speed was readjusted by ±0.1 km/h. Upon completing the adjustment, the subject stepped down from the treadmill and rested for approximately 2 min before climbing onto the treadmill again. They were then asked to walk on the treadmill for approximately 30–60 s and, once they assumed a stable walking pattern, treadmill walking data were collected for 1 min. Gait tests were conducted at the preferred speed, 20% faster than the preferred speed, and finally, 20% slower than the preferred speed. Then, the participants rested for approximately 1 min between each speed (Figure 2).

### 2.4. Data Analysis and Statistical Analysis

All statistical analyses were performed using SPSS 21.0 (IBM, Inc., Armonk, NY, USA). The Shapiro–Wilk test was used to examine whether data were normally distributed. A sample independent t-test was performed to analyze the mean and standard deviation for the age and sex groups. The post-hoc test was performed using ANCOVA after controlling for the body mass index (BMI), number of falls, MMSE, IPAQ-SF scores, and age (only sex groups) between the groups (60–69 years group compared with 70–79 years group, and males compared with females). Additionally, the responsiveness between the groups 60–69 years and 70–79 years and that between males and females was expressed as the effect size (ES). Effect sizes were interpreted as small (<0.50), medium (0.50–0.79), or large (≥0.80) as previously described [37]. Prior to additional analysis, Z-normalization (value-mean/standard deviation) was performed to normalize all variables. Stepwise binary logistic regression analysis was performed to determine the classifiers of age-specific groups and sex-specific groups. Differences with *p* < 0.05 were considered to be significant.

The gait data were collected at a sampling frequency of 100 Hz, and filtered to a cut-off frequency of 10 Hz using a second-order Butterworth low-pass filter [32,33]. The heel strikes and toe-offs of gait events were detected when the linear acceleration along the anteroposterior and vertical axes were maximum, respectively [32,33]. GV was calculated using the coefficient of variance (CV; standard deviation/mean × 100) to measure the variability of the stride length, time, and single and double support and stance phases. Gait asymmetry (GA) and phase coordination index (PCI) were calculated using the approach used in [36]. GA was evaluated by comparing the swing time required by each leg, and PCI was calculated based on a combination of the percentage_ABS_φ and CV of φ [36].

## 3. Results

### 3.1. Demographic and Physical Characteristics of the Participants

Table 1 shows the demographic, physical characteristics, and three walking speeds of the 225 participants included in the study. In the age comparison, males aged 70–79 years had significantly higher age (*p* < 0.001) and lower diastolic blood pressure (*p* = 0.018) than those aged 60–69 years. Females aged 70–79 years had significantly higher age (*p* < 0.001) and systolic blood pressure (*p* = 0.021) and lower walking speeds (slower speed, *p* = 0.006; preferred speed, *p* = 0.013; faster speed, *p* = 0.014) than those aged 60–69 years. In the sex comparison, males aged 60–69 years had significantly higher age (*p* = 0.014), height (*p* < 0.001), body mass (*p* < 0.001), diastolic blood pressure (*p* = 0.042), physical activity (*p* < 0.001), education (*p* < 0.001), walking speeds (slower speed, *p* = 0.034; preferred speed, *p* = 0.031; faster speed, *p* = 0.048), lower body fat percentage (*p* < 0.001) and number of falls (*p* = 0.006) than females aged 60–69 years. Males aged 70–79 years had significantly higher height (*p* < 0.001), body mass (*p* < 0.001), physical activity (*p* < 0.001), education (*p* = 0.009), walking speeds (slower speed, *p* < 0.001; preferred speed, *p* = 0.001; faster speed, *p* = 0.001), and lower body fat percentage (*p* < 0.001) than females aged 60–69 years.

### 3.2. Group Differences: 60s vs. 70s and Males vs. Females

An analysis of the results based on age revealed that the males aged 70–79 years exhibited a significantly higher CV of the double support phase than those aged 60–69 years (preferred speed, ES = 0.057, *p* = 0.032). The females aged 70–79 years exhibited significantly higher CVs of the stride length (slower speed, ES = 0.039, *p* = 0.021; preferred speed, ES = 0.073, *p* = 0.001; faster speed, ES = 0.085, *p* = 0.001), stride time (slower speed, ES = 0.039, *p* = 0.021; preferred speed, ES = 0.073, *p* = 0.001; faster speed, ES = 0.085, *p* = 0.001), single support phase (slower speed, ES = 0.063, *p* = 0.003; preferred speed, ES = 0.064, *p* = 0.003; faster speed, ES = 0.070, *p* = 0.002), stance phase (slower speed, ES = 0.029, *p* = 0.048; preferred speed, ES = 0.052, *p* = 0.008; faster speed, ES = 0.067, *p* = 0.002), and GA (preferred speed, ES = 0.036, *p* = 0.027) than those aged 60–69 years.

Analysis of the results based on sex revealed that the females aged 60–69 years exhibited a significantly higher CVs of the stride length (faster speed, ES = 0.046, *p* = 0.022), stride time (faster speed, ES = 0.046, *p* = 0.022), stance phase (faster speed, ES = 0.065, *p* = 0.006), and GA (preferred speed, ES = 0.036, *p* = 0.042) than the males of the same age group. The females aged 70–79 years exhibited significantly higher GA (preferred speed, ES = 0.057, *p* = 0.016; faster speed, ES = 0.052, *p* = 0.022) than the males of the same age group (Table 2).

### 3.3. Classifier Variables for Age-Specific Groups and Sex-Specific Groups

Stepwise binary logistic regression analysis of the male groups of 60–69 and 70–79 years revealed that the CV of the double support phase at the preferred speed differed significantly (odds ratio (OR): 1.658, 95% confidence interval (CI): 1.035–2.655, *p* = 0.036). Similarly, for females of the same age groups, CV of the stride length at faster speed (OR: 2.176, 95% CI: 1.361–3.481, *p* = 0.001) and GA at the preferred speed (OR: 1.526, 95% CI: 1.048–2.222, *p* = 0.027) differed significantly (Table 3). An analysis of the male and female groups of 60–69 years revealed significant differences in the CV of the stance phase at the faster speed (OR: 0.443, 95% confidence interval (CI): 0.223–0.882, *p* = 0.020) and GA values at preferred speed (OR: 0.532, 95% CI: 0.302–0.936, *p* = 0.029) differed significantly. In addition, for 70–79 years groups, GA value at preferred speed (OR: 1.998, 95% CI: 1.152–3.464, *p* = 0.014) differed significantly (Table 4).

## 4. Discussion

The main findings of this study are summarized as follows: (1) Males aged 70–79 years exhibited a higher CV of the double support phase at the preferred speed conditions than those aged 60–69 years, and the CV could distinguish the elderly males by age. (2) Females aged 70–79 years exhibited higher CVs of the stride length and time, single support phase, and stance phase at all three speed conditions along with higher GA values at the preferred speed than those aged 60–69 years. Moreover, the CV of the stride length at the faster speed and the GA at the preferred speed could distinguish the elderly females by age. (3) Males aged 60–69 years exhibited higher CVs of the stride length and time, stance phase at the faster speed, and GA at the preferred speed than females of the same age group, and CV of the stance phase at the faster speed and the GA at the preferred speed could distinguish ages 60–69 years by sex. (4) Females aged 70–79 years exhibited a higher GA at the preferred and faster speeds than males of the same age group, and the GA at the preferred speed could distinguish ages 70–79 years by sex.

In previous studies involving treadmill walking analysis based on various speeds, GV was reported to be associated with an increase in age due to the CVs of stride and step length and stride time [38,39]. Similarly, our study revealed that a higher CV of the double support phase at the preferred speed was associated with an increase in the age of the male participants, and these differences were associated with relatively small ESs (d = 0.057). The higher CVs of stride length and time, single support phase, and stance phase at all three speed conditions along with higher GA values at the preferred speed were associated with an increase in the age of the female participants. These differences were associated with relatively small ESs (d = 0.029–0.085). Elderly gait patterns such as slower walking speed, longer double support and stance phases, and shorter single support phase can contribute to an increase in the GV parameters GA and PCI, indicating a decline in gait stability [13,31,36] that might be related to reduced muscle strength and flexibility [38] and decreased neurotransmitter ability owing to muscle and proprioceptive degeneration with aging [39].

Interestingly, our logistic regression analysis results indicated that the CV of the double support phase at the preferred speed was approximately 65.8% higher in males aged 70–79 years than that in ages 60–69 years. It has been reported previously that the CV of the double support phase is associated with the dynamic balance during gait [9], and is dependent on proprioceptive feedback to maintain consistent timing in the double support phase [18]. In addition, females aged 70–79 years had a higher CV of the stride length at the faster speed (an approximately 117.6% increase) and GA at the preferred speed (an approximately 52.6% increase) than females aged 60–69 years. These results might indicate that gait characteristics differ with the ability to adapt to speed changes on a tread-mill. In previous studies, the CV of the stride length reflected control of the gait-related rhythm stepping mechanism [9], and depends on a central pattern generator in the basal ganglia and spinal cord that produces gait automaticity [10,40]. This automaticity enables a low degree of GV that requires minimal attention [10], whereas a high degree of GV requires high-level motor cortical control and attention [8]. Thus, the increase in GV in the elderly may be due to a degeneration of the basal ganglia and central nervous system with aging [8].

The GV at the preferred speed of elderly individuals in terms of variables, such as the CVs of step length, step width, stride time, double support time, and stance time, is reported to be significantly higher in females than in males [41,42]. Recently, Johansson et al. [43] reported that elderly males had significantly lower CVs of stride and step length, double support time, swing time, and stance time at both preferred and faster speeds and lower CVs of stride and step time at faster speeds than elderly females. However, our results for sex comparisons males aged 60–69 years had significantly higher CVs of the stride length and time, stance phase at the faster speed, and GA at the preferred speed than females of the same age group. These differences were associated with relatively small ESs (d = 0.036–0.065). In addition, the results of our logistic regression analysis indicated that the CV of the stance phase at the faster speed and the GA at the preferred speed is a variable that distinguishes ages 60–69 years by sex. Previous studies reported increased step widths and double support phases in enhancing the dynamic stability of elderly adults during walking, and these changes evoked longer stance phases in response to the reduced lower-limb strength [1,30,44,45]. In our study, the stance phase values at the faster speed showed no significant differences between sexes in ages 60–69 years (male = 60.31 ± 1.55, female = 60.64 ± 1.27). However, the CV of the stance at the faster speed showed significant differences, and it was a major variable that could distinguish the sexes among ages 60–69 years. Thus, these results suggested that the faster speed condition was a more challenging task for males aged 60–69 years. Future studies that include challenging gait tasks considering the sex rate are needed to explore this sex difference in identifying the GV among elderly.

Furthermore, females aged 70–79 years had significantly higher GA at the preferred and faster speeds than males of the same age group. These differences were associated with relatively small ESs (d = 0.052–0.057). In addition, the GA at the preferred speed was indicated as a variable that distinguishes ages 70–79 years by sex. GA is an indicator of the degree of asymmetry between left and right steps, and increases in this value have been reported to reduce bilateral coordination. This may be associated with worsened gait coordination ability and dynamic stability [36,46]. As reported previously [5], gait disorders tend to be more prevalent in females than in males after the age of 70. These results might reflect an increased prevalence of joint pain, degradation of muscle strength and muscle reactivity, and various other changes involved in the advanced aging of females [5,42]. However, we did not consider the factors related to muscle strength or joint pain in the analysis in this study. Therefore, it is suggested that the relationship between these factors should be confirmed in future studies.

Walking slower or faster than the preferred speed reduces energy storage and recovery; it can consume more mechanical energy due to the alteration of gait and lower limb muscle movements [22,47]. Slower walking speeds might also involve the use of a strategy to increase the mediolateral displacement of the center of mass to maintain dynamic balance and increase the base of support [48], whereas faster walking speeds might require lower limb muscle functioning to increase joint motion range, propulsion, and dynamic stability to increase step length [47,49]. Therefore, under objective and varied speed conditions, we suggest that it can be difficult for elderly individuals to maintain the symmetry needed to ensure dynamic stability. This modification of gait speed can potentially reduce gait automaticity by high attentional motor cortex control patterns [30,31] and force reliance on high-level execution functions that require additional cognitive load, thereby degrading the processing and modification of gait [50,51]. Song and Geyer [52] suggested that the integration of sensory feedback in undertaking tasks at slower and faster walking speeds was more important functionally than the central pattern generator of the spinal cord. We therefore suggest that gait analysis using a treadmill under various speed conditions is an appropriate method for evaluating GV of elderly individuals.

Our study confirmed the GV characteristics of the participants according to age and sex, and confirmed the characteristics of the reduction in gait ability according to the detailed age for each sex. Furthermore, our study showed significant results for various speed conditions along with the preferred speed, and suggested that diversity of tasks is needed in gait analysis among the elderly. However, this study had several limitations. First, this was conducted as a pilot study with a limited sample size and no follow-up. Therefore, future studies should include more participants to expand and segment age categories and minimize the dropout effect. Second, our results cannot exclude the possibility of sampling bias for sex, because there were more females than males. Finally, we recruited participants with prior experience in using treadmills. The participants were asked to walk naturally on the treadmill, as in daily life, to the greatest extent possible during the testing procedure. Some previous studies have reported no significant differences in the spatiotemporal parameters between treadmill walking and over-ground walking in healthy humans [23,24,25,26]. However, treadmill walking may reduce the variance of steps when compared with over-ground walking, because the treadmill mechanically regulates the walking speed and constrains the participants to walk along a straight line [24]. In addition, the treadmill walking speed acts as an external cue for the participants [31]. Nevertheless, all the participants completed the treadmill walking tasks successfully at all the speed conditions, and this may be utilized as a reference in gait training programs.

## 5. Conclusions

In this study, we analyzed the GV characteristics of elderly individuals while walking on a treadmill for one minute at three speed conditions to distinguish between age groups and sex. An analysis of the results by age groups revealed that the CV of the double support phase at the preferred speed could distinguish the elderly males by age group. Variables such as the CV of the stride length at faster speed and the GA at the preferred speed could distinguish the elderly females from the males by age group. In addition, an analysis of the results by sex groups revealed that the CV of the stance phase at the faster speed and GA at the preferred speed could distinguish the sex of the elderly aged 60–69 years. The GA at the preferred speed could distinguish the sex of the elderly individuals aged 70–79 years. Specifically, it was shown that the GV data obtained during the treadmill walking test were useful in determining the change in gait characteristics according to the age and sex of the elderly individuals who participated in the study. The elderly may need physical activity programs [53] and regular walking activities [54] to prevent falls because an increase in GV leads to an increased risk of falls due to reduced balance [16]. Therefore, we suggest that it is necessary to enroll in treadmill walking intervention programs using various speeds to improve the motor function of the elderly and reduce the potential risk that would lead to the decline of their gait ability.

In the future, we aim to conduct experiments to analyze the relationship between the decline in nerve function and gait variability by segmenting the age range and expanding the number of elderly participants.

## Figures and Tables

**Table 1:** Demographic and physical characteristics of the participants.

Variables		Males (*n* = 85)	Females (*n* = 140)	Age			Sex		
					*p* Value	d		*p* Value	d
Age (years)	60s	66.64 ± 2.05	65.55 ± 2.41	M	<0.001	3.006	60s	0.014	0.479
	70s	73.72 ± 2.62	72.70 ± 2.73	F	<0.001	2.791	70s	0.057	0.380
Height (cm)	60s	168.90 ± 4.74	154.82 ± 4.59	M	0.077	0.388	60s	<0.001	3.033
	70s	166.96 ± 5.21	153.49 ± 5.21	F	0.109	0.274	70s	<0.001	2.586
Body mass (kg)	60s	70.39 ± 7.07	58.40 ± 6.20	M	0.121	0.340	60s	<0.001	1.839
	70s	67.56 ± 9.40	59.45 ± 7.55	F	0.367	0.154	70s	<0.001	0.971
BMI (kg/m^2^)	60s	24.68 ± 2.36	24.36 ± 2.37	M	0.510	0.143	60s	0.492	0.132
	70s	24.26 ± 3.38	25.25 ± 3.07	F	0.058	0.325	70s	0.122	0.308
BFP (%)	60s	24.00 ± 5.16	34.01 ± 5.80	M	0.312	0.221	60s	<0.001	1.792
	70s	25.20 ± 5.66	34.32 ± 6.05	F	0.753	0.054	70s	<0.001	1.547
SBP (mmHg)	60s	131.48 ± 16.55	128.70 ± 13.72	M	0.916	0.037	60s	0.329	0.188
	70s	132.02 ± 12.73	134.35 ± 15.40	F	0.021	0.390	70s	0.382	0.162
DBP (mmHg)	60s	85.48 ± 8.28	82.01 ± 9.04	M	0.018	0.543	60s	0.042	0.394
	70s	80.60 ± 9.59	82.57 ± 10.54	F	0.821	0.057	70s	0.382	0.193
MMSE (score)	60s	28.07 ± 1.92	27.43 ± 1.76	M	0.363	0.198	60s	0.067	0.354
	70s	27.67 ± 2.08	27.71 ± 2.00	F	0.371	0.153	70s	0.921	0.020
IPAQ-SF (MET-min/week)	60s	5215.56 ± 4679.71	3141.58 ± 2000.03	M	0.735	0.074	60s	<0.001	0.647
	70s	5507.12 ± 3106.27	3070.56 ± 2969.02	F	0.867	0.029	70s	<0.001	0.805
Education (years)	60s	12.36 ± 2.88	9.13 ± 2.54	M	0.057	0.419	60s	<0.001	1.213
	70s	11.00 ± 3.56	9.44 ± 2.50	F	0.464	0.125	70s	0.009	0.523
Number of falls (N)	60s	0.02 ± 0.15	0.30 ± 0.63	M	0.131	0.331	60s	0.006	0.533
	70s	0.14 ± 0.47	0.19 ± 0.53	F	0.281	0.184	70s	0.614	0.100
Walking speed (m/s)									
Slower speed	60s	0.88 ± 0.17	0.82 ± 0.11	M	0.997	0.006	60s	0.034	0.412
	70s	0.88 ± 0.16	0.76 ± 0.17	F	0.006	0.472	70s	<0.001	0.715
Preferred speed	60s	1.10 ± 0.21	1.03 ± 0.13	M	0.891	0.030	60s	0.031	0.418
	70s	1.09 ± 0.20	0.96 ± 0.19	F	0.013	0.426	70s	0.001	0.672
Faster speed	60s	1.31 ± 0.25	1.23 ± 0.16	M	0.982	0.005	60s	0.048	0.383
	70s	1.31 ± 0.25	1.15 ± 0.23	F	0.014	0.423	70s	0.001	0.676

All data represent the mean ± standard deviation; 60s—aged 60–69 years; 70s—aged 70–79 years; M—males; F—females; BMI—body mass index; BFP—body fat percentage; SBP—systolic blood pressure; DBP—diastolic blood pressure; MMSE—mini mental state examination; IPAQ-SF—International Physical Activity Questionnaire-short form; MET—metabolic equivalents; d—Cohen’s d (small = <0.50, medium = 0.50–0.79, large = ≥0.80); significant difference = *p* < 0.05.

**Table 2:** Comparison of gait variability characteristics by age and sex.

Variables		Slower Speed		Preferred Speed		Faster Speed		Age Group Significance		Sex Group Significance	
		Males	Females	Males	Females	Males	Females	Males	Females	60s	70s
CV of stride length (%)	60s	2.61 ± 1.12	2.81 ± 1.09	1.86 ± 0.74	1.81 ± 0.52	1.55 ± 0.46	1.47 ± 0.44	N/S	A, B, C	G	N/S
	70s	2.90 ± 1.33	3.25 ± 1.09	1.94 ± 0.68	2.15 ± 0.73	1.71 ± 0.75	1.81 ± 0.61				
CV of stride time (%)	60s	2.61 ± 1.12	2.81 ± 1.09	1.86 ± 0.74	1.81 ± 0.52	1.55 ± 0.46	1.47 ± 0.44	N/S	A, B, C	G	N/S
	70s	2.90 ± 1.33	3.25 ± 1.09	1.94 ± 0.68	2.15 ± 0.73	1.71 ± 0.75	1.81 ± 0.61				
CV of single support phase (%)	60s	5.39 ± 2.40	5.43 ± 1.71	3.57 ± 1.47	3.42 ± 0.97	2.77 ± 0.95	2.65 ± 0.75	N/S	A, B, C	N/S	N/S
	70s	5.31 ± 2.01	6.36 ± 1.97	3.43 ± 1.23	3.99 ± 1.22	2.85 ± 0.96	3.13 ± 0.84				
CV of double support phase (%)	60s	9.23 ± 4.30	8.20 ± 2.87	6.12 ± 2.03	6.17 ± 1.92	5.98 ± 2.07	5.53 ± 1.40	B	N/S	N/S	N/S
	70s	9.26 ± 3.89	9.21 ± 3.62	7.07 ± 2.27	6.52 ± 1.77	6.14 ± 1.96	5.84 ± 1.59				
CV of stance phase (%)	60s	4.01 ± 1.80	4.10 ± 1.34	2.62 ± 1.06	2.43 ± 0.64	2.04 ± 0.68	1.85 ± 0.51	N/S	A, B, C	G	N/S
	70s	4.13 ± 1.75	4.65 ± 1.60	2.67 ± 0.85	2.83 ± 1.04	2.17 ± 0.84	2.23 ± 0.81				
GA	60s	2.80 ± 2.06	2.98 ± 2.14	2.46 ± 1.47	2.09 ± 1.36	1.90 ± 1.29	1.84 ± 1.09	N/S	B	F	F, G
	70s	2.84 ± 2.14	3.59 ± 2.62	1.89 ± 1.29	2.81 ± 2.09	1.51 ± 1.14	2.27 ± 1.81				
PCI	60s	4.70 ± 1.61	4.91 ± 2.03	3.62 ± 1.42	3.92 ± 1.35	3.36 ± 2.00	3.60 ± 1.71	N/S	N/S	N/S	N/S
	70s	4.89 ± 1.82	5.32 ± 2.38	3.72 ± 1.20	4.02 ± 1.45	3.37 ± 1.18	3.67 ± 1.86				

All data represent the mean ± standard deviation; CV—coefficient of variance; GA—gait asymmetry; PCI—phase coordination index; 60s—aged 60–69 years; 70s—aged 70–79 years; group differences between 60s and 70s for slower (A), preferred (B), and faster (C) speeds, model adjusted for BMI, number of falls, MMSE score, and IPAQ-SF score; group differences between males and females for slower (E), preferred (F), and faster (G) speeds, model adjusted for BMI, number of falls, MMSE score, IPAQ-SF score, and age; *p* < 0.05; N/S indicates no significance.

**Table 3:** Binary logistic regression results for 60–69 years and 70–79 years.

Variables	Estimate	SE	OR	95% CI for OR	*p* Value	R_N_^2^
Male						
P_CV of double support phase (%)	0.505	0.240	1.658	1.035–2.655	0.036	0.128
Female						
F_CV of stride length (%)	0.778	0.240	2.176	1.361–3.481	0.001	0.217
P_GA	0.423	0.192	1.526	1.048–2.222	0.027	

Dependent variable—age (0 = aged 60∼69, 1 = aged 70∼79); model adjusted for BMI, number of falls, MMSE score, and IPAQ-SF score; CV—coefficient of variance; GA—gait asymmetry; P—preferred speed; F—faster speed; SE—standard error; OR—odds ratio; CI—confidence interval; significant difference = *p* < 0.05.

**Table 4:** Binary logistic regression results for males and females.

Variables	Estimate	SE	OR	95% CI for OR	*p* Value	R_N_^2^
Aged 60~69						
F_CV of stance phase (%)	−0.813	0.351	0.443	0.223–0.882	0.020	0.461
P_GA	−0.632	0.289	0.532	0.302–0.936	0.029	
Aged 70~79						
P_GA	0.692	0.281	1.998	1.152–3.464	0.014	0.349

Dependent variable—sex (0 = male group, 1 = female group); model adjusted for BMI, number of falls, MMSE score, IPAQ-SF score, and age; GA—gait asymmetry; P—preferred speed; SE—standard error; OR—odds ratio; CI—confidence interval; significant difference = *p* < 0.05.

## Data Availability

The datasets generated and/or analyzed during the current study are not publicly available due to intellectual property reasons but are available upon a reasonable request.
